# Transcranial Direct Current Stimulation in the Treatment of Gait Disturbance in Post-Stroke Patients: An Overview of Systematic Reviews

**DOI:** 10.3390/s23239301

**Published:** 2023-11-21

**Authors:** Juan Antonio Chamorro-Hinojosa, Francisco Molina-Rueda, María Carratalá-Tejada

**Affiliations:** 1 International Doctorate School, Rey Juan Carlos University, 28008 Madrid, Spain; ja.chamorro.2020@alumnos.urjc.es; 2 Department of Physical Therapy, Occupational Therapy, Rehabilitation and Physical Medicine, Faculty of Health Sciences, Rey Juan Carlos University, 28922 Alcorcón, Spain; maria.carratala@urjc.es

**Keywords:** physiotherapy, neurorehabilitation, transcranial electrical stimulation, stroke, gait, non-invasive stimulation techniques

## Abstract

Introduction: Transcranial direct current stimulation (tDCS) is a promising technique for brain modulation after a cerebrovascular accident (CVA). This treatment modality has been previously studied in the recovery of patients. The aim of this review is to analyse the evidence for the application of tDCS in the recovery of gait disturbance in stroke patients. Methods: This review was conducted according to the recommendations of the PRISMA statement. Three different electronic databases were searched for relevant results: PubMed, Scopus, and Cochrane, from 2015 to January 2022. We included reviews and meta-analyses that only considered randomised controlled trials (RCTs) that investigated the effects of transcranial direct current stimulation, in combination or not with other physiotherapy treatments, compared to no treatment, usual care, or alternative treatment on gait recovery. Our primary outcomes of interest were walking speed, mobility, and endurance; secondary outcomes included motor function. Results: Thirteen studies with a total of 195 RCTs were included. Data on population, outcome measures, protocols, and outcomes were extracted. The Amstar-2 scale and the GRADE system of certainty of evidence were used. Only one study received high certainty of evidence, 5 received low certainty of evidence, and 7 received critically low certainty of evidence. Moderate to low-quality evidence showed a beneficial effect of tDCS on gait parameters, but not significantly. Conclusions: Although the tDCS produces positive changes in gait recovery in spatio-temporal parameters such as mobility, endurance, strength, and motor function, there is insufficient evidence to recommend this treatment. Higher-quality studies with larger sample sizes are needed for stronger conclusions.

## 1. Introduction

Stroke is one of the leading causes of disability in the world, and most survivors have limitations in mobility and gait [1]. Gait performance is considered an indicator of health and quality of life in people who have suffered a stroke [2], because gait performance is linked to the development of activities of daily living and community participation. Therefore, it is considered one of the most important objectives in the recovery of stroke survivors [3,4].

In recent years, numerous techniques have attempted to address gait impairments in stroke survivors using different approaches. Some of these techniques, such as percutaneous nerve electrostimulation, virtual reality, biofeedback using electromyography, neurophysiological approaches, or the use of auditory signals during gait re-education, have been classified by the American Stroke Association as level IIb-B evidence, indicating an unestablished recommendation and conflicts in evidence [5].

Non-invasive brain stimulation (NIBS) includes several techniques developed to achieve neuromodulation of the brain. It has its origins in the 1980s with the application of current to healthy subjects, which was a revolution compared to direct brain stimulation [6]. Two main techniques are described: magnetic fields and electrical stimulation. Transcranial electrical stimulation (TES) involves the application of a weak electrical current to the scalp, powered by batteries and connected to an anode and a cathode. Its effect lies in the modulation of the polarisation of cell membranes, and it is voltage-dependent [7,8]. The most popular type of TES is the tDCS [8]. The long-term effects of transcranial electrical stimulation are commonly defined by two concepts: long-term potentiation (LTP), related to neural excitation, and long-term depression (LTD), related to neural inhibition [9]. Both, which are synaptic mechanisms of plasticity, are related to the stimulation protocol; thus, the anode is more related to LTP effects, while the cathode is more related to LTD effects [10]. Their application is usually 1–2 mA, and the duration of the treatment is between 10 and 30 min [11].

The key to the application of these techniques is the durability of the effects after treatment, which are associated with repeated stimulation in short periods of time, increasing the benefits and prolonging neuroplasticity. However, although the application of tDCS has a modulating effect at the cortical level that seems to favour the patient’s recovery and is associated with benefits in certain pathologies, there is a lack of evidence to support the use of these techniques for the recovery of gait in neurological patients [5]. This could be due to possible differences in application protocols, as factors such as electrode size, connector position, and the conductivity of the different electrode materials, including saline concentration and conductive gel, could affect the results obtained [7].

Several reviews have attempted to answer the evidence of tDCS application for gait recovery in stroke patients [4,12,13,14,15,16,17,18,19,20,21,22,23,24]. However, the variability of the protocols and the heterogeneity of the outcome measures make it difficult to interpret the results. Other authors have attempted to synthesise and address the evidence for these protocols for the recovery of upper limb function [25]. However, no systematic review and meta-analyses for gait recovery has been conducted to allow the generation of evidence-based tables and clinical recommendations. Therefore, an overview of systematic reviews and meta-analyses is needed to summarise the effect of the application of tDCS in post-stroke patients for gait recovery through the description of evidence-based tables that facilitate the clinical recommendation of the different applications of this technique using the GRADE method.

## 2. Materials and Methods

### 2.1. Database Search Strategy

The following databases were searched on 21 March 2023: PubMed, Scopus, and Cochrane Database of Systematic Reviews. The search terms used were: “stroke”, “cerebrovascular accident”, “apraxia of gait”, “gait disorder”, and “gait dysfunction”. A combination of these and similar terms was performed using the Boolean operators AND and OR (Table 1). We did not search the grey literature, as we aimed for studies that had been previously screened by reviewers. We also did not consult with experts in the field; conference abstracts, dissertations, or reports were not published on personal websites. The review protocol was registered with PROSPERO (CRD42021237915).

### 2.2. Eligibility Criteria

- Type of studies: This study only includes systematic reviews (with or without meta-analysis) that analysed randomised controlled trials due to their methodological quality. Published from 2015 to 2022. Only titles written in English and Spanish were considered.
- Types of participants:
  ○ Subjects diagnosed with an ischaemic or haemorrahagic stroke.
  ○ Adults over 18 years of age.
  ○ Acute, sub-acute, or chronic stroke.
- Type of intervention: we included studies in which the interventions involved tDCS, whether administered alone or in combination with another form of treatment and compared to another form of physical therapy, or placebo. We excluded papers that did not define interventions, multi-therapies, or any pharmacological treatment (e.g., botulinum toxin).
- Types of outcome measures: studies quantitatively assessing gait pattern (three-dimensional instrumental analysis systems), gait speed, functional mobility, endurance, motor function, and muscle strength.

### 2.3. Study Selection and Data Extraction

The method of data collection was based on the Cochrane Handbook of Systematic Reviews of Interventions. Review Manager 5 software (RevMan 2020) was used for review writing [26].

Two reviewers screened the title, abstract, and full texts independently for the inclusion of all systematic reviews potentially identified in the search. Differences were resolved by discussion or, if necessary, the consultation of a third team member.

Two authors independently extracted data from the reviews using a predefined data extraction form. Where outcome information was unclear or missing, the individual published file was accessed for further details.

### 2.4. Methodological Assessment and Evaluation of the Quality of Evidence

No reassessment was performed on the studies included in the reviews; however, the quality of the studies was reported according to the assessment of the author of each review. This information was subtracted during data collection and extraction.

The methodological quality of each systematic review was assessed using AMSTAR 2 (A Messurement Tool to Assess Systematic Review) [27]. Based on 16 items, this tool assesses the methodology used in a systematic review. Each item is rated as “yes” (clearly conducted), “no” (clearly not conducted), “cannot be answered”, or “not applicable”. Finally, the studies are classified according to their confidence. “High”, the systematic review has no weaknesses. “Medium”, no critical weaknesses but some non-critical weaknesses. “Low” has some critical weaknesses. “Critically Low” has more than one critical weakness.

We assessed the quality of evidence for our primary endpoint, the evidence for the use of tDCS for gait training in stroke patients, using GRADE. We used the recommendation methods described in chapter 12, section 8.a of the Cochrane handbook [28], using GRADEpro GDT [software 2021]. All decisions to downgrade the quality of studies were justified by comments so that the reader could understand the decisions and agree or disagree.

## 3. Results

### 3.1. Selection of Studies

**Figure 1:**
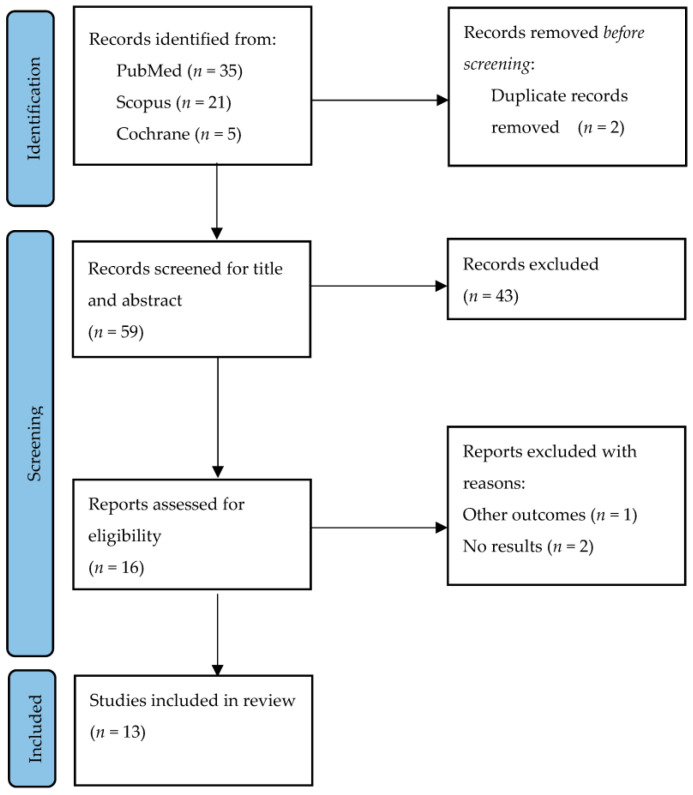
Flow chart (PRISMA).

A total of 47 studies were found, and duplicates were removed, leaving a total of 21 articles. Studies were screened by title and abstract to check that they met the previously established inclusion criteria. Finally, 13 reviews were included [4,12,13,14,15,18,19,20,21,22,23,24,29] for a total of 195 randomised controlled trials (RCT). The selection process is shown in the flow chart (Figure 1) with a list of excluded studies, reasons, and funding (Table S1).

### 3.2. Methodological Quality Assessment

The results of the methodological assessment of the included systematic reviews/meta-analyses are shown in Table 2. Only one study was rated with “High” confidence [12], although it did not justify this study design decision. While 7 studies were rated with “Critically Low” confidence [4,14,18,20,21,22,29], the remaining 5 studies were classified with “Low” confidence [13,15,19,23,24].

### 3.3. Assessing the Quality of Evidence

The results of the evidence assessment of the included systematic reviews/meta-analyses are shown in Table S1. The following outcomes of interest were broken down: gait speed, functional mobility, endurance, motor function, and muscle strength.

### 3.4. Summary of Results

The characteristics of the included studies are summarised in Table 3. Of the 13 included studies, we found 8 meta-analyses [4,12,14,18,20,21,22,29] and 5 systematic reviews [13,15,19,23,24]. All articles defined the intervention as applying tDCS in combination with another physiotherapy treatment (treadmill, walking assistance robot, conventional physiotherapy, etc.) or alone. The control group received a placebo stimulation treatment, in combination or not with physiotherapy, or a different stimulation protocol.

The outcome measures described by the reviews were organised as follows: spatiotemporal parameters of gait [4,13,14,15,18,19,20,21,23,24,29] functional mobility [4,13,14,15,18,19,20,21,23,24] gait endurance [4,13,15,18,19,20,21,23,24] motor function [17,19,24,29] muscle strength [4,12,13,14,24] and lower limb functionality [12,22].

#### 3.4.1. Effect of tDCS in Combination with Physiotherapy on Spatiotemporal Parameters

The outcome measures described for this outcome were 10 MWT (10 Meter walk test), quantitative analysis using technological systems, and gait cadence. For gait speed, the meta-analyses by Li et al. [4], Vaz et al. [14], Tien et al. [18], and Dong et al. [21] found no significant improvements in their meta-analyses. Mitsutake et al. [20] included both online and offline stimulation in their analysis. Only the online application showed a significant improvement over offline. According to GRADE, the certainty of the evidence for these studies was classified as “low” and “very low”.

As for systematic reviews, Paz et al. [13] and Santos et al. [19] expressed significant improvements in walking speed (9.09%, *p* = 0.046). For Navarro-López et al. [15] and Bressi et al. [24], there were no significant improvements in any of the included studies. For the review by Corominas-Teruel et al. [23], the results were presented as effect sizes. Only one study showed a large effect in this respect. According to GRADE, they were classified as having “low” and “very low” certainty (Table S1).

As for gait cadence, which was recorded as steps per minute (p/m), Vaz et al. [14] and Mitsutake et al. [20] showed no significant differences. According to GRADE, they were classified with “very low” certainty of evidence (Table S1) [4,13,14,15,18,19,20,21,22,23,24].

#### 3.4.2. Effect of tDCS in Combination with Physiotherapy on Functional Mobility

The outcome measures were the TUG (Time up and go), the FAC (Functional ambulatory category), the RMI (Rivermead mobility index), and the Tinetti test. In the studies by Vaz et al. [14], Mitsutake et al. [20], and Dong et al. [21], no significant improvements were found in their meta-analyses evaluated by FAC and TUG. Whereas Tien et al. [18], who found significant improvements in describing FAC, RMI, and TUG, found no significant differences with the Tinetti test. According to GRADE, the certainty of the evidence was classified as “low” and “very low”. However, Li et al. [4], who were the only ones with a “moderate” certainty of evidence, showed significant improvements for functional mobility.

In terms of systematic reviews, studies find controversial results depending on the outcome measures used. It is noted that Paz et al. [13], Navarro-López et al. [15], Bressi et al. [24], and Santos et al. [19] do not provide sufficient evidence to recommend the use of tDCS for functional mobility. For the review by Corominas-Teruel et al. [23], who evaluated the results using cohen’s d, also did not consider there was sufficient evidence for a treatment recommendation.

According to GRADE, except for Paz et al. [13], which classified the certainty of the evidence as moderate, the rest were classified as “low” and “very low” (Table S2).

#### 3.4.3. Effect of tDCS in Combination with Physiotherapy on Endurance

The outcome measure for this outcome was the 6 MWT (6 Minute walk test). Li et al. [4], Tien et al. [18], and Dong et al. [21] reported no significant improvements in their meta-analyses. Mitsutake et al. [20] included both online and offline applications in their analysis. Only the online application showed significant improvements over the offline application. According to GRADE, the certainty of the evidence was classified as “low” and “very low” [4,18,20,21].

In terms of systematic reviews, the included studies show controversial results. Paz et al. [13], Navarro-López et al. [15], Santos et al. [19], and Bressi et al. [24] were not considered to show significant improvements in gait endurance. Corominas-Teruel et al. [23], who evaluated the results using cohen’s d, also did not have enough evidence to make a treatment recommendation. According to GRADE, the certainty of the evidence was classified as “low” and “very low” (Table S2).

#### 3.4.4. Effect of tDCS in Combination with Placebo on Motor Function

The outcome measure used for this outcome was the FMA-LE (Fugl-Meyer assessment lower extremity). Dong et al. [21] and Li et al. [16] reported no significant improvement. According to GRADE, the certainty of the evidence was classified as “low”.

The systematic review by Paz et al. [13], Navarro-López et al. [15], Santos et al. [19], and Bressi et al. [24] was not considered to have enough evidence to recommend the use of tDCS to improve motor function. According to GRADE, the certainty of the evidence was classified as “very low” (Table S2).

#### 3.4.5. Effect of tDCS in Combination with Physiotherapy on Muscle Strength

The outcome measures for this outcome were the MRC (Medical Research Council) and MI-LE (Motricity Index Lower Extremity). Although Li et al. [4] reported significant improvements, Vaz et al. [14] did not report significant improvements in muscle strength. According to GRADE, the certainty of the evidence was classified as “low” and “very low”.

In terms of systematic reviews, only Paz et al. [13] and Bressi et al. [24] reported insufficient evidence to recommend tDCS treatment for muscle strength. The certainty of the evidence was classified as “low” (Table S2).

#### 3.4.6. Effect of tDCS in Combination with Placebo on Lower Limb Function

Outcome measures for this outcome were spatiotemporal parameters (10 MWT and cadence), endurance (6 MWT), functional mobility (FAC, Tinetti, and MI-LE), motor function (FMA), and balance (BBS). Elsner et al. [12] showed no significant improvements in either statistical analysis. Veldema et al. [22] performed a comparative meta-analysis of different stimulation protocols. They described a large effect on the application of tDCS in the contralateral and bilateral hemispheres. The certainty of the evidence was classified as “low” and “very low” (Table S1) [12,22].

## 4. Discussion

This review has included 8 meta-analyses and 5 systematic reviews with the aim of assessing the quality of evidence regarding the application of tDCS in the recovery of gait in post-ACV patients. The evidence has been assessed using the AMSTAR-2 tool for risk of bias and GRADEpro for certainty of evidence. Outcome measures for lower limb recovery were extracted and classified as follows: spatiotemporal parameters (gait speed and cadence), functional mobility, endurance, muscular strength, motor function, and lower limb functionality.

Although some studies have shown that tDCS improves gait speed [20], functional mobility [4,13,18], muscle strength [4], endurance [20], and functionality of the lower limbs [22] compared to sham stimulation and the control group, the certainty of the evidence does not seem homogeneous to recommend the benefits of the application of tDCS.

Brain reorganisation after stroke seems to be crucial to patient recovery [30]. However, it has been observed that the healthy hemisphere may influence this reorganisation during the recovery period. Abnormalities are observed in the interhemispheric relationship to produce voluntary movement [31] and the reduced excitability of the injured hemisphere, particularly the primary motor cortex (M1), compared to the intact hemisphere [32]. Some theories also point to the influence of the intact hemisphere on affect, but this cannot be proven. Therefore, although tDCS techniques appear to be a useful tool for influencing factors related to corticospinal excitability of the injured hemisphere and recovery, the results seem to be inconclusive.

Previous authors have reviewed the evidence for these techniques in upper limb recovery [33,34], but there does not seem to be clear evidence of their influence on functional recovery. Whereas other authors have shown significant results in the application of tDCS for the prevention of falls and recovery of the lower limb [35], this may be due to differences in the brain representation of the upper limbs compared to the lower limbs. It has been observed that the activation of mechanically similar joints does not follow the same patterns as would be expected; in fact, it is related to the task being performed. For example, in a brain image, we can see that the contraction of the elbow follows a more lateral pattern, while the contraction of the knee follows a more medial pattern [36]. Therefore, tDCS techniques may give different results depending on the task targeted for recovery, as reflected in this gait study.

In this review, we found a large heterogeneity of findings suggesting that the application of tDCS could lead to improvements in gait, but not all results seem to be conclusive. This could be explained by differences in application protocols or sample size, as most of them include a small number of participants, even in combination with several studies. As for the outcome measures, there is a high heterogeneity in the management and statistical analysis of these data.

On the other hand, it is important to mention the differences in the application protocols for the different techniques. Regarding the application of tDCS, the intensity applied in the included studies ranges between 1 and 2.5 mA, although other authors have previously set the threshold for corticospinal excitation at 2 mA for the anode [37]. Another important factor is the application of unilateral or bilateral tDCS. Most of the included reviews involve studies where unilateral application (anode or cathode) is predominant for excitatory or inhibitory purposes. Although the benefit of unilateral application in post-ACV patients has been demonstrated [38], some reviews continue to include RCTs that perform bilateral application [4,15,18]. Finally, some studies differentiate between online delivery, which combines stimulation and some targeted activity simultaneously, and offline delivery, which performs pre-treatment stimulation. No direct benefits are observed for offline stimulation [20].

This overview of systematic reviews and meta-analyses presents an update on the evidence on the application of tDCS for gait recovery. However, these results should be interpreted with caution due to some limitations in the review process. First, only studies published in any journal that were in English and/or Spanish were included. Second, an exhaustive search of the grey literature was not conducted. Third, the sample size in the included studies is too small to draw robust conclusions. Fourth, some reviews include studies with a high risk of bias, especially in the randomisation and blinding of patients, therapists, and assessors. Fifth, the high heterogeneity in the studies included must be considered.

## 5. Conclusions

Our overview provides a comprehensive picture of the use of tDCS in clinical practise among stroke survivors. The different protocols used in the trials attempted to stimulate brain reorganisation for neuroplasticity. Nevertheless, the best stimulation protocol in terms of time and electrode positioning is not yet clear.

Due to the quality of the literature and the statistical results, this review suggests that no firm recommendation can be made for or against the use of tDCS in combination or not with other therapies. Although the trials suggest possible positive effects on patients’ recovery compared with the control group and especially the placebo group, the low quality of the trials and the low certainty of the evidence, together with the fact that most of the reviews showed no significant differences between the groups, led the authors to make a conditional recommendation for intervention or comparison.

## Figures and Tables

**Table 1:** Full search strategy.

**PubMed Search**
(((((((((((((stroke[Title/Abstract]) OR (cva[Title/Abstract])) OR (“acquired brain injury”[Title/Abstract])) OR (cerebral stroke[MeSH Terms])) AND (tDCS[Title/Abstract])) OR (“transcranial direct current stimulation”[Title/Abstract])) OR (NIBS[Title/Abstract])) OR (“non-invasive brain stimulation”[Title/Abstract])) AND (physical therapy[Title/Abstract])) OR (placebo[Title/Abstract])) AND (gait[Title/Abstract])) OR (“gait disorder”[Title/Abstract])) OR (apraxia of gait[MeSH Terms])) OR (“lower extremity function”[Title/Abstract]) AND ((meta-analysis[Filter] OR systematicreview[Filter]) AND (2016:2022[pdat]))
**Cochrane Search**
“transcranial direct current stimulation”:ti,ab,kw AND “stroke”:ti,ab,kw
**Scopus Search**
stroke AND tdcs AND gait AND PUBYEAR > 2014 AND PUBYEAR < 2023 AND (LIMIT TO (DOCTYPE, “re”)) AND (LIMIT TO (SUBAREA, “HEAL”))

**Table 2:** AMSTAR checklist.

AMSTAR Checklist	Li et al. [4]	De Paz et al. [13]	Vaz et al. [14]	Elsner et al. [12]	Tien et al. [18]	Santos et al. [19]	Mitsutake et al. [20]	Dong et al. [21]	Navarro-López et al. [15]	Veldema et al. [22]	Corominas-Teruel et al. [23]	Bressi et al. [24]
**1**	Y	Y	Y	Y	Y	Y	Y	N	Y	Y	Y	Y
**2**	N	N	Y	Y	N	Y	Y	Y	N	N	Y	Y
**3**	N	N	N	N	N	N	N	N	N	N	N	N
**4**	PY	PY	PY	Y	N	PY	PY	PY	Y	PY	PY	PY
**5**	Y	Y	Y	Y	Y	Y	Y	N	Y	Y	Y	Y
**6**	Y	N	Y	Y	Y	Y	Y	N	Y	Y	Y	Y
**7**	N	N	N	Y	N	N	N	N	PY	N	N	N
**8**	Y	Y	Y	Y	Y	Y	Y	PY	Y	Y	Y	Y
**9**	Y	Y	Y	Y	Y	Y	Y	Y	Y	Y	Y	Y
**10**	N	N	N	Y	N	N	N	N	N	N	N	N
**11**	Y	NA	Y	Y	Y	NA	Y	Y	NA	Y	NA	NA
**12**	Y	NA	N	Y	Y	NA	Y	Y	NA	Y	NA	NA
**13**	Y	Y	Y	Y	Y	Y	Y	Y	Y	Y	Y	Y
**14**	Y	Y	Y	Y	Y	Y	Y	Y	Y	Y	Y	Y
**15**	N	NA	N	Y	N	NA	N	N	NA	N	NA	NA
**16**	N	Y	Y	Y	Y	Y	Y	Y	Y	Y	Y	Y
**Overall assessment**	Critically low	Low	Critically low	High	Critically low	Low	Critically low	Critically low	Low	Critically low	Low	Low

1 = Question PICO; 2 = Protocol registered prior to review; 3 = Justification of the design of the studies included; 4 = adequate literature search; 5 = Selection of duplicate studies; 6 = Duplicate data extraction; 7 = Justification of excluded studies; 8 = Adequate description of the studies included; 9 = Risk of bias in individual included studies; 10 = source of funding for the studies included; 11 = Appropriate meta-analytical methods; 12 = Risk of bias assessment in meta-analysis; 13 = Consideration of the risk of bias in the interpretation of the results of the review; 14 = Explanation of heterogeneity in results; 15 = Assessment of the presence and impact of publication bias; 16 = Conflict of interest. Y = Yes; N = No; PY = Partial Yes; NA = Not Applicable.

**Table 3:** Summary of included studies.

Review	Data Assessed as up to Date	Population	Interventions	Comparison Interventions	Outcomes for Which Data Were Reported	Review Limitations
Li et al. (2018) [4]	April 2017 (English)	Post-stroke patients over 18 years of age.	**tDCS**: Transcranial direct current stimulation is applied to the anode in the ipsilateral (affected) hemisphere (*n* = 6). Combined therapy with physiotherapy, robotic-assisted therapy, and task-oriented training. Applying cathode to the contralateral hemisphere (*n* = 2). Rehabilitation treatment. Bihemispheric application (*n* = 3). Rehabilitation treatment.	Simulated treatment (complementary robot-assisted treatments, task-related training, robotic orthoses, and conventional rehabilitation).	Gait speed (10 MWT) and quantitative gait analysis (GAITRite and Eagle Digital System) Functional mobility (TUG, Tinetti, and FAC) Running resistance (6 MWT) Strengths of MMII (Medical Research Council and MI-LE)	It only included studies in one language (English). Heterogeneity in outcome measures and tDCS application parameters. Low number of studies. Low sample size. Some authors did not cooperate with the data request, and the data may be biased.
De Paz et al. (2019) [13]	2018	Patients diagnosed with a pathology of the central nervous system.	**tDCS** applying anode in ipsilateral (affected) hemisphere (*n* = 4).	Simulated treatment of tDCS. No intervention. Conventional motor rehabilitation treatment.	Walking speed (10 MWT) Functional mobility (TUG, FAC, and FMA) Resistance (6 MWT) MMII strength (MI-LE and MRC)	Heterogeneity in tDCS application parameters. Low number of studies. Low sample size. Only articles in English and Spanish. It does not include data on comparator groups. Misquoted references.
Vaz et al. (2019) [14]	December 2018	Subjects who have suffered an acute/subacute (less than six months) or chronic (more than six months) stroke.	**tDCS**: Transcranial direct current stimulation is applied to the anode in the ipsilateral (affected) hemisphere (*n* = 6). Applying cathode contralateral hemisphere (*n* = 1). **rTMS** (*n* = 3)	Simulated treatment of tDCS rTMS combined or not with other types of therapy (conventional, robotic, treadmill, motor imagery)	Walking speed (10 MWT) Functional mobility (FAC) MMII strength (MI) Cadence	Heterogeneity in tDCS application parameters. Low number of studies. The sample size of the included studies was low. Heterogeneity in the exercises described as “conventional therapy” in the control group.
Elsner et al. (2020) [12]	January 2019 (all languages)	Post-stroke patients over 18 years of age.	**tDCS**: Transcranial direct current stimulation is applied to the anode in the ipsilateral (affected) hemisphere (*n* = 7). Applying cathode to the contralateral hemisphere (*n* = 2). Bihemispheric application (*n* = 2).	Simulated treatment of tDCS No intervention Conventional motor rehabilitation treatment	MMII function (gait speed, FAC) Muscular strength	Heterogeneity in the pooling of results in meta-analysis. Heterogeneity in comparison therapy. Low sample size.
Tien et al. (2020) [18]	January 2019	Post-stroke patients over 18 years of age.	**tDCS**: tDCS applying cathode to the affected hemisphere (*n* = 1). tDCS applying anode on affected hemisphere (*n* = 9). Bihemispheric application (*n* = 3).	Simulated treatment of tDCS.	Walking speed (10 MWT) Functional gait mobility (FAC, Tinetti, TUG, and RMI) Resistance (6 MWT)	Low sample size. Heterogeneity in application protocols. Heterogeneity in comparison therapy. Only articles in English and Chinese.
Santos et al. (2020) [19]	October 2018	Children, adolescents, adults, and older people who do not have a progressive central nervous system disease.	**tDCS** in combination with motor training.	Simulated toeatment in combination with motor training.	Walking speed (10 MWT) Functional mobility (TUG) Resistance (6 MWT) Motor function (FMA)	Low sample size. Heterogeneity in application protocols. Articles are in English and Portuguese only.
Matsutake et al. (2021) [20]	19 March 2021	Patients diagnosed with haemorrhagic or ischaemic stroke with unilateral hemiplegia. They can walk without support and can maintain their weight and balance.	**tDCS**: Anode in affected hemisphere + combined therapy simultaneously (online) (*n* = 4). Anode in the affected hemisphere followed by combined therapy (not simultaneously) (offline) (*n* = 2).	Simulated tDCS treatment in combination with robot-assisted gait therapy or neuromuscular stimulation.	Walking speed (10 MWT) Functional Gait Mobility (FAC and TUG) Resistance (6 MWT) Cadence (gait analysis)	Low sample size. Heterogeneity in application protocols. Heterogeneity in comparison therapy. Studies are published in English only.
Dong et al. (2021) [21]	August 2020	Patients who have been diagnosed with a stroke.	**tDCS** applying anode in ipsilateral hemisphere.	Simulated treatment of tDCS.	Walking speed (10 MWT) Functional gait mobility (FAC, TUG and FMA-LE) Resistance (6 MWT)	Low sample size. Heterogeneity in application protocols. Heterogeneity in comparison therapy. Studies published in English only.
Navarro-López et al. (2021) [15]	March 2020	Patients who have been diagnosed with a stroke.	**tDCS**: tDCS applying cathode to the affected hemisphere (*n* = 2). tDCS applying anode on affected hemisphere (*n* = 6). Bihemispheric application (*n* = 4).	Simulated treatment of tDCS.	Walking speed (10 MWT) Functional gait mobility (FAC, TUG, and Tinetti) Resistance (6 MWT)	Low sample size. Heterogeneity in application protocols. Heterogeneity in comparison therapy. Studies are published only in English and Spanish.
Veldema et al. (2022) [22]	31 March 2021	Patients diagnosed with stroke.	**NIBS**: tDCS rTMS iTBS tACS tsDCS	Simulated stimulation	Lower limb functionality (combining outcome measures of balance, gait and motor function).	Low sample size Low number of studies Only included studies in English or German.
Corominas-Teruel et al. (2022) [23]	7 February 2022	Patients who have suffered a stroke aged 18 years or older.	**tDCS**.	Simulated stimulation alone or in combination with other therapies.	Walking speed Functional mobility (TUG) Resistance (6 MWT)	Heterogeneity in application parameters Only includes studies in English
Bressi et al. (2022) [24]	15 March 2021	Patients over 18 years of age who have suffered a stroke in a chronic process (>6 months).	**tDCS** in combination with gait-assisted robot.	Simulated stimulation	Walking speed (10 MWT) Functional mobility (TUG, RMI, and FAC) Resistance (6 MWT) Motor function (FMA) Muscle strength (MRC)	Heterogeneity in application parameters Low number of studies Low sample size Only included studies in English Includes all types of studio designs

tDCS = Transcranial Direct-Current Stimulation; rTMS = repetitive transcranial magnetic stimulation; NIBS = Non-invasive brain stimulation; iTBS = intermitent transcranial brain stimulation; tACS = transcranial alterning current stimulation; tsDCS = transcranial spinal direct current stimulation ; FAC = Functional Ambulatory Category; TUG = Time Up and Go; FMA = Fugl Meyer Assessment; 6 MWT = 6 min walking test; 10 MWT = 10 m walking test; MAS = Modified Ashworth Scale; RMI = Rivermead Mobility Index; CT = conventional therapy; MI-LE = Lower limb Motricity Index.

## Data Availability

Not applicable.

## Supplementary Materials

The following supporting information can be downloaded at: https://www.mdpi.com/article/10.3390/s23239301/s1. Table S1: Excluded studies; Table S2: Summary of the quality of evidence (GRADE).
