# Supplementary material for: Transcranial Direct Current Stimulation in the Treatment of Gait Disturbance in Post-Stroke Patients: An Overview of Systematic Reviews
Source: Sensors (Basel). 2023 Nov 21;23(23):9301. doi: 10.3390/s23239301 (PMC10708691; doi:10.3390/s23239301)
Supplement: Supplementary file 1 [file sensors-23-09301-s001.zip › sensors-2694785-supplementary.pdf]

Table S1. Excluded studies.

Excluded items	Reasons	Source of funding
The Effects of Transcranial Direct Current Stimulation (tDCS) on Balance Control in Older Adults: A Systematic Review and Meta-Analysis. **Guo et al.**	Other outcomes	DB was supported by Key Research and Development Projects of the Ministry of Science and Technology (2018YFC2000602); BM and JZ were supported by a Hebrew Senior Life Marcus-Applebaum pilot grant, as well as grants from the National Institutes of Health (R21AG064575; R01AG059089-01), the Boston Claude D. Pepper Older Americans Independence Center (P30-AG013679), and the Boston Roybal Center for Active Lifestyle Interventions (P30AG048785).
**Non-invasive brain stimulation in rehabilitation** Serdar Kesikburun	No results	The authors received no financial support for this research and/or authorship of this article.
Transcranial Direct Current Stimulation for Motor Recovery Following Brain Injury April Pruski and Gabriela Cantarero	No results	No sources of funding are indicated.

Table S2. Summary of the quality of evidence (GRADE).

Summary of results: Li et al. [4]

tDCS combined with physiotherapy compared to placebo for improving gait in post-stroke patients

Patient or population: improving gait in post-stroke patients
Configuration:
Intervention: *tDCS* combined with physiotherapy
Comparison: placebo

Outcomes	**Anticipated absolute effects*** (95% CI)		Relative effect (95% CI)	Number of participants (studies)	Certainty of evidence (GRADE)	Comments
	**Placebo risk**	**Risk with tDCS combined with physiotherapy**				
Gait speed was evaluated with: *10MWT* and Quantitative analysis	-	SMD is **0.39 SD higher.** (0.06 lower to 0.85 higher).	-	79 (5 Randomised Controlled Trials (RCTs))	⊕⊕◯◯ Low[a,b]	No significant differences are found between groups favouring the use of *tDCS* to improve walking speed.
Functional mobility is assessed with: *TUG*, *FAC,* and *Tinetti*	-	SMD is **0.44 SD higher.** (0.01 higher. to 0.87 higher.)	-	89 (5 Randomised Controlled Trials (RCTs))	⊕⊕⊕◯ Moderate[b]	Significant between-group differences are found in favour of *tDCS* in combination with physiotherapy to improve functional mobility.
Resistance was evaluated with: *6MWT*	-	SMD is **0.28 SD higher.** (0.24 lower than 0.84 higher).	-	51 (3 Randomised Controlled Trials [RCTs])	⊕⊕◯◯ Low[a,b]	No significant differences are found between groups favouring the use of *tDCS* to improve gait endurance.
Muscle strength is assessed with: *MI-LE* and *MRC*	-	SMD is **1.54 SD higher.** (0.29 higher. to 2.78 higher.)	-	84 (3 Randomised Controlled Trials [RCTs])	⊕⊕◯◯ Low[b,c]	Significant differences are found in favour of using *tDCS* in combination with physiotherapy to improve muscle strength.

The risk in the intervention group (and its 95% confidence interval) is based on the risk assumed in the comparison group and the **relative effect** of the intervention (and its 95% confidence interval).

CI: Confidence Interval; **SMD:** Standardised Mean Difference.

Summary of results: Li et al. [4]

tDCS combined with physiotherapy compared to placebo for improving gait in post-stroke patients

Patient or population: improving gait in post-stroke patients
Configuration:
Intervention: *tDCS* combined with physiotherapy
Comparison: placebo

Outcomes	**Anticipated absolute effects*** (95% CI)		Relative effect (95% CI)	Number of participants (studies)	Certainty of evidence (GRADE)	Comments
	**Placebo risk**	**Risk with tDCS combined with physiotherapy**				

GRADE Working Group Degrees of Evidence
High certainty: We are very confident that the true effect is close to the effect estimate.
Moderate certainty: We have moderate confidence in the effect estimate: the true effect is likely to be close to the effect estimate, but there is a possibility that it could be substantially different.
Low certainty: We have limited confidence in the effect estimate: the actual effect may be substantially different from the effect estimate.
Very low certainty: We have very low confidence in the effect estimate: The actual effect is likely to be substantially different from the effect estimate.

Explanations

a. The CI crosses the clinical decision threshold between recommending and not recommending treatment.
b. Low sample size
c. High inconsistency due to heterogeneity > 80% and low CI overlap.

Summary of results: De Paz et al. [13]

tDCS combined with physiotherapy compared to a placebo for improving gait in post-stroke patients

Patient or population: improving gait in post-stroke patients
Configuration:
Intervention: *tDCS* combined with physiotherapy
Comparison: placebo

Outcomes	Anticipated absolute effects* (95% CI)		Relative effect (95% CI)	Number of participants (studies)	Certainty of evidence (GRADE)	Comments
	**Placebo risk**	**Risk with *tDCS* combined with physiotherapy**				
Walking speed was evaluated with: *10MWT*	1 study reported a reduction in test time of almost 10% (9.09%, p=0.046). 2 studies showed no significant difference.			67 (3 Randomised Controlled Trials [RCTs])	⨁⨁◯◯ Low[a,b,c]	There is no clear evidence on the effect of *tDCS* on walking speed.
Functional mobility assessed is with: *TUG* and *FAC*	1 study showed a reduction in *TUG* time of 5.29% (p=0.026), and a final study showed an increase in FAC mobility at the 2nd assessment of 44.5% (p=0.03). One study showed no significant difference.			91 (4 Randomised Controlled Trials [RCTs])	⨁⨁⨁◯ Moderate[b,c]	There is no clear evidence on the effect of *tDCS* on functional mobility.
Resistance evaluated with: *6MWT*	1 study showed an increase in resistance at assessment 3 of 60.35% (p=0.038). Another study showed no significant difference.			37 (2 Randomised Controlled Trials [RCTs])	⨁⨁◯◯ Low[a,b,c]	There is no clear evidence on the effect of *tDCS* on gait resistance.
Muscle strength assessed is with: *MRC* and *MI-LE*	1 study reported an increase in muscle strength in *MI-LE* of 6.9% (p=0.031). 2 studies showed no significant difference.			60 (3 Randomised Controlled Trials [RCTs])	⨁⨁◯◯ Low[a,b,c]	There is no clear evidence on the effect of *tDCS* on muscle strength.
Motor Functionality is assessed with: *FMA-LE*	A single study reported an increase in *AMF* of 6.27% (p=0.023).			(1 RCT (randomised controlled experiment))	⨁⨁⨁◯ Moderate[b,c]	*tDCS* combined with physiotherapy is likely to result in a slight increase in motor function.

The risk in the intervention group (and its 95% confidence interval) is based on the risk assumed in the comparison group and the **relative effect** of the intervention (and its 95% confidence interval).

CI: Confidence Interval

Summary of results: De Paz et al. [13]

tDCS combined with physiotherapy compared to a placebo for improving gait in post-stroke patients

Patient or population: improving gait in post-stroke patients
Configuration:
Intervention: *tDCS* combined with physiotherapy
Comparison: placebo

Outcomes	**Anticipated absolute effects*** (95% CI)		Relative effect (95% CI)	Number of participants (studies)	Certainty of evidence (GRADE)	Comments
	**Placebo risk**	**Risk with *tDCS* combined with physiotherapy**				

GRADE Working Group Degrees of Evidence
High certainty: We are very confident that the true effect is close to the effect estimate.
Moderate certainty: We have moderate confidence in the effect estimate: the true effect is likely to be close to the effect estimate, but there is a possibility that it could be substantially different.
Low certainty: We have limited confidence in the effect estimate: the actual effect may be substantially different from the effect estimate.
Very low certainty: We have very low confidence in the effect estimate: The actual effect is likely to be substantially different from the effect estimate.

Explanations

a. The direction and magnitude of the effect vary across studies.
b. Low sample size.

Summary of results: Vaz et al. [14]

tDCS combined with physiotherapy compared to a placebo for improving gait in post-stroke patients

Patient or population: improving gait in post-stroke patients
Configuration:
Intervention: *tDCS* combined with physiotherapy
Comparison: placebo

Outcomes	**Anticipated absolute effects*** (95% CI)		Relative effect (95% CI)	Number of participants (studies)	Certainty of evidence (GRADE)	Comments
	**Placebo risk**	**Risk with tDCS combined with physiotherapy**				
Walking speed was evaluated with: *10MWT*	The average walking speed was **0.03** m/s.	MD **0.02 m/s higher.** (0.08 lower to 0.12 higher.)	-	134 (7 Randomised Controlled Trials (RCTs))	⊕◯◯◯ Very low[a,b,c]	No significant differences are found between groups favouring the use of *tDCS* to improve walking speed.
Functional mobility assessed is with: *FAC* Scale from: 0 to 5	The average functional mobility was **0.64**	MD **0.13 higher.** (0.31 lower to 0.57 higher).	-	104 (6 Randomised Controlled Trials [RCTs])	⊕◯◯◯ Very low[a,b,c,d]	No significant differences are found between groups in favour of the use of *tDCS* to improve functional mobility.
Muscle strength is assessed with: *MI-LE*	The average muscle strength was **11.2**	MD **1.66 higher.** (1.72 lower than 5.03 higher.)	-	64 (3 Randomised Controlled Trials [RCTs])	⊕◯◯◯ Very low[b,c,e]	No significant differences are found favouring the use of *tDCS* to improve muscle strength.
Cadence evaluated with: Steps per minute	The average cadence was **7.1** steps per minute.	MD **3.19 steps per minute higher.** (6.72 lower to 13.09 higher.)	-	156 (3 Randomised Controlled Trials [RCTs])	⊕◯◯◯ Very low[b,c,f]	No significant differences were found favouring the use of *tDCS* to improve gait cadence.

The risk in the intervention group (and its 95% confidence interval) is based on the risk assumed in the comparison group and the **relative effect** of the intervention (and its 95% confidence interval).

CI: Confidence Interval; **MD:** Mean Difference

GRADE Working Group Degrees of Evidence
High certainty: We are very confident that the true effect is close to the effect estimate.
Moderate certainty: We have moderate confidence in the effect estimate: the true effect is likely to be close to the effect estimate, but there is a possibility that it could be substantially different.
Low certainty: We have limited confidence in the effect estimate: the actual effect may be substantially different from the effect estimate.
Very low certainty: We have very low confidence in the effect estimate: The actual effect is likely to be substantially different from the effect estimate.

Explanations

a. 60% of the studies were reported to be of low methodological quality. Three studies had unclear randomised sequencing; others had a lack of blinding in randomisation and blinding of participants and assessors.
b. A low sample size is evident.
c. The CI crosses the clinical decision threshold between recommending and not recommending treatment.
d. Differences in effect estimates are observed. CIs do not overlap.
e. 2/3 of the studies have a low methodological quality.
f. Poor methodological quality in the most important study.

Summary of results: Elsner et al. [12]

tDCS combined with physiotherapy compared to placebo for improving gait in post-stroke patients

Patient or population: improving gait in post-stroke patients
Configuration:
Intervention: *tDCS* combined with physiotherapy
Comparison: placebo

Outcomes	**Anticipated absolute effects*** (95% CI)		Relative effect (95% CI)	Number of participants (studies)	Certainty of evidence (GRADE)	Comments
	**Placebo risk**	**Risk with *tDCS* combined with physiotherapy**				
MMII functionality at the end of treatment (absolute values) evaluated with: *10MWT, 6MWT, and FAC*	-	SMD is **0.28 SD higher.** (0.12 lower to 0.69 higher).	-	204 (8 Randomised Controlled Trials (RCTs))	⊕⊕◯◯ Low[a,b]	No significant differences were found in favour of the use of *tDCS* to improve lower limb function.
MMII functionality at the end of treatment (change values) evaluated with: *10MWT, 6MWT, and FAC*	-	SMD is **0.46 SD higher.** (0.09 lower to 1.01 higher).	-	54 (4 Randomised Controlled Trials [RCTs])	⊕⊕◯◯ Low[a,b]	No significant differences were found in favour of the use of *tDCS* to improve lower limb function.

The risk in the intervention group (and its 95% confidence interval) is based on the risk assumed in the comparison group and the **relative effect** of the intervention (and its 95% confidence interval).

CI: Confidence Interval; **SMD:** Standardised Mean Difference.

Summary of results: Elsner et al. [12]

tDCS combined with physiotherapy compared to placebo for improving gait in post-stroke patients

Patient or population: improving gait in post-stroke patients
Configuration:
Intervention: *tDCS* combined with physiotherapy
Comparison: placebo

Outcomes	**Anticipated absolute effects*** (95% CI)		Relative effect (95% CI)	Number of participants (studies)	Certainty of evidence (GRADE)	Comments
	**Placebo risk**	**Risk with *tDCS* combined with physiotherapy**				

GRADE Working Group Degrees of Evidence
High certainty: We are very confident that the true effect is close to the effect estimate.
Moderate certainty: We have moderate confidence in the effect estimate: the true effect is likely to be close to the effect estimate, but there is a possibility that it could be substantially different.
Low certainty: We have limited confidence in the effect estimate: the actual effect may be substantially different from the effect estimate.
Very low certainty: We have very low confidence in the effect estimate: The actual effect is likely to be substantially different from the effect estimate.

Explanations

a. Low sample size.
b. The CI overpasses the threshold between recommending and not recommending a treatment.

Summary of results: Tien et al. [18]

tDCS combined with physiotherapy compared to a placebo for improving gait in post-stroke patients

Patient or population: improving gait in post-stroke patients
Configuration:
Intervention: *tDCS* combined with physiotherapy
Comparison: placebo

Outcomes	**Anticipated absolute effects*** (95% CI)		Relative effect (95% CI)	Number of participants (studies)	Certainty of evidence (GRADE)	Comments
	**Placebo risk**	**Risk with *tDCS* combined with physiotherapy**				
Gait speed was evaluated with: *10MWT* and quantitative	-	SMD is **0.189 SD higher.** (0.135 lower than 0.513 higher).	-	154 (7 Randomised Controlled Trials (RCTs))	⊕◯◯◯ Very low[a,b,c]	There is no significant difference in favour of using *tDCS* to improve walking speed.
Functional mobility is assessed with: *FAC*	-	SMD is **0.542 SD higher.** (0.142 higher. to 0.942 higher.)	-	108 (5 Randomised Controlled Trials [RCTs])	⊕⊕◯◯ Low[a,b]	Significant differences in favour of the use of *tDCS for* the improvement of functional mobility are evident.
Functional mobility is assessed with: *Tinetti*	-	SMD is **0.441 SD higher.** (0.022 lower to 0.904 higher).	-	75 (3 Randomised Controlled Trials [RCTs])	⊕◯◯◯ Very low[a,b,c]	No significant differences are evident in favour of the use of *tDCS* to improve functional gait mobility.
Functional mobility is assessed with: *RMI*	-	SMD is **0.699 SD higher.** (0.18 higher. to 1.219 higher.)	-	62 (3 Randomised Controlled Trials [RCTs])	⊕⊕◯◯ Low[a,b]	Significant differences in favour of the use of *tDCS for* the improvement of functional mobility are evident.
Functional mobility is assessed with: *TUG*	-	SMD is **0.676 SD higher.** (0.293 higher. to 1.058 higher.)	-	84 (5 Randomised Controlled Trials [RCTs])	⊕⊕◯◯ Low[a,b]	Significant differences in favour of the use of tDCS for improving functional mobility are evident.
Resistance evaluated with: *6MWT*	-	SMD is **0.209 SD higher.** (0.338 lower to 0.756 higher).	-	52 (3 Randomised Controlled Trials [RCTs])	⊕◯◯◯ Very low[a,b,c]	No significant differences are evident in favour of the use of tDCS to improve functional gait mobility.

The risk in the intervention group (and its 95% confidence interval) is based on the risk assumed in the comparison group and the **relative effect** of the intervention (and its 95% confidence interval).

CI: Confidence Interval; **SMD:** Standardised Mean Difference.

Summary of results: Tien et al. [18]

tDCS combined with physiotherapy compared to a placebo for improving gait in post-stroke patients

Patient or population: improving gait in post-stroke patients
Configuration:
Intervention: *tDCS* combined with physiotherapy
Comparison: placebo

Outcomes	**Anticipated absolute effects*** (95% CI)		Relative effect (95% CI)	Number of participants (studies)	Certainty of evidence (GRADE)	Comments
	**Placebo risk**	**Risk with *tDCS* combined with physiotherapy**				

GRADE Working Group Degrees of Evidence
High certainty: We are very confident that the true effect is close to the effect estimate.
Moderate certainty: We have moderate confidence in the effect estimate: the true effect is likely to be close to the effect estimate, but there is a possibility that it could be substantially different.
Low certainty: We have limited confidence in the effect estimate: the actual effect may be substantially different from the effect estimate.
Very low certainty: We have very low confidence in the effect estimate: The actual effect is likely to be substantially different from the effect estimate.

Explanations

a. The included studies have hidden allocation biases.
b. Low sample size (<400)
c. The CI exceeds the threshold between recommending and not recommending treatment.

Summary of results: Santos et al. [19]

tDCS combined with motor training compared to placebo to improve gait in post-stroke patients

Patient or population: improving gait in post-stroke patients
Configuration:
Intervention: *tDCS* combined with motor training
Comparison: placebo

Outcomes	Anticipated absolute effects* (95% CI)		Relative effect (95% CI)	Number of participants (studies)	Certainty of evidence (GRADE)	Comments
	**Placebo risk**	**Risk with *tDCS* combined with motor training**				
Walking speed was evaluated with: *10MWT*	4 studies reported no significant differences in favour of the experimental group. Only 1 study showed significant differences in favour of the use of *tDCS*.			109 (5 Randomised Controlled Trials (RCTs))	⨁◯◯◯ Very low[a,b,c]	There is no clear evidence on the effect of tDCS on walking speed.
Functional mobility is assessed with: *TUG*	1 study showed significant differences in favour of the use of *tDCS*. Another study found no significant differences between treatments.			39 (2 Randomised Controlled Trials [RCTs])	⨁◯◯◯ Very low[a,b,c]	There is no clear evidence on the effect of tDCS on functional mobility.
Gait resistance was tested with: *6MWT*	2 studies reported that the experimental group was significantly larger than the control group ($p<0.05$). 1 study showed no significant difference between groups.			107 (3 Randomised Controlled Trials [RCTs])	⨁◯◯◯ Very low[a,b,c,d]	There is no clear evidence on the effect of tDCS on resistance.
Motor function is assessed with: FMA	1 study showed significant differences between groups in favour of the experimental group. Another study showed no difference in the use of *tDCS*.			54 (2 Randomised Controlled Trials [RCTs])	⨁◯◯◯ Very low[a,b,c]	There is no clear evidence on the effect of tDCS on motor function.
Spatio-temporal parameters of gait	1 study evaluated the parameters stride length, stride length, and cadence, but found no significant differences between groups.			24 (1 RCT (randomised controlled experiment))	⨁◯◯◯ Very low[a,b,c]	There is no evidence on the effect of *tDCS* on the improvement of spatio-temporal parameters.

The risk in the intervention group (and its 95% confidence interval) is based on the risk assumed in the comparison group and the **relative effect** of the intervention (and its 95% confidence interval).

CI: Confidence Interval

Summary of results: Santos et al. [19]

tDCS combined with motor training compared to placebo to improve gait in post-stroke patients

Patient or population: improving gait in post-stroke patients
Configuration:
Intervention: *tDCS* combined with motor training
Comparison: placebo

Outcomes	**Anticipated absolute effects*** (95% CI)		Relative effect (95% CI)	Number of participants (studies)	Certainty of evidence (GRADE)	Comments
	**Placebo risk**	**Risk with *tDCS* combined with motor training**				

GRADE Working Group Degrees of Evidence
High certainty: We are very confident that the true effect is close to the effect estimate.
Moderate certainty: We have moderate confidence in the effect estimate: the true effect is likely to be close to the effect estimate, but there is a possibility that it could be substantially different.
Low certainty: We have limited confidence in the effect estimate: the actual effect may be substantially different from the effect estimate.
Very low certainty: We have very low confidence in the effect estimate: The actual effect is likely to be substantially different from the effect estimate.

Explanations

a. Risk of bias in blinding during randomisation.
b. Low sample size (<400)
c. Narrative synthesis, vague.
d. The direction of effect varies across studies.

Summary of results: Mitsutake et al. [20]

tDCS combined with physiotherapy compared to placebo improved gait in post-stroke patients

Patient or population: improving gait in post-stroke patients
Configuration:
Intervention: *tDCS* combined with physiotherapy
Comparison: placebo

Outcomes	Anticipated absolute effects* (95% CI)		Relative effect (95% CI)	Number of participants (studies)	Certainty of evidence (GRADE)	Comments
	**Placebo risk**	**Risk with tDCS combined with physiotherapy**				
Online running speed was tested with: *10MWT*	-	SMD is **0.48 SD higher.** (0.01 higher. to 0.94 higher.)	-	73 (3 Randomised Controlled Trials [RCTs])	⊕⊕◯◯ Low[a,b]	Significant differences in favour of the use of online *tDCS* to improve gait speed in post-stroke patients are evident.
Offline running speed was tested with: *10MWT*	-	SMD is **0.08 SD higher.** (0.41 lower to 0.58 higher.)	-	62 (2 Randomised Controlled Trials [RCTs])	⊕⊕◯◯ Low[b,c]	There is no significant difference in favour of the use of offline *tDCS for* the improvement of gait speed in post-stroke patients.
Total walking speed was evaluated with: *10MWT*	-	SMD is **0.29 SD higher.** (0.05 lower to 0.64 higher.)	-	135 (5 Randomised Controlled Trials [RCTs])	⊕◯◯◯ Very low[b,c,d]	There is no significant difference in favour of the use of *tDCS for* the improvement of walking speed in post-stroke patients.
Functional mobility is assessed with: *FAC*	-	SMD is **0 SD** (1.82 lower to 1.81 higher.)	-	41 (2 Randomised Controlled Trials [RCTs])	⊕◯◯◯ Very low[b,c,e,f]	There is no significant difference in favour of the use of *tDCS* for functional gait speed mobility in post-stroke patients.
Functional mobility is assessed with: *TUG*	-	SMD is **0.26 SD higher.** (0.2 less than 0.73 higher.)	-	71 (2 Randomised Controlled Trials [RCTs])	⊕◯◯◯ Very low[b,c,e]	There is no significant difference in favour of the use of *tDCS* for functional gait speed mobility in post-stroke patients.
Online gait resistance was tested with: *6MWT*	-	SMD is **1.08 SD higher.** (0.42 higher. to 1.77 higher.)	-	40 (2 Randomised Controlled Trials [RCTs])	⊕⊕◯◯ Low[b,e]	Significant differences are evident in favour of the use of online *tDCS* to improve gait endurance in post-stroke patients.
Offline gear resistance	-	SMD is **0.25 SD lower** (0.76 lower than 0.25 higher.)	-	62 (2 Randomised Controlled Trials [RCTs])	⊕⊕◯◯ Low[b,c]	There is no significant difference in favour of the use of offline *tDCS for* gait speed endurance in post-stroke patients.

Summary of results: Mitsutake et al. [20]

tDCS combined with physiotherapy compared to placebo improved gait in post-stroke patients

Patient or population: improving gait in post-stroke patients
Configuration:
Intervention: *tDCS* combined with physiotherapy
Comparison: placebo

Outcomes	Anticipated absolute effects* (95% CI)		Relative effect (95% CI)	Number of participants (studies)	Certainty of evidence (GRADE)	Comments
	**Placebo risk**	**Risk with tDCS combined with physiotherapy**				
Total running resistance was tested with: *6MWT*	-	SMD is **0.4 SD higher.** (0.38 lower than 1.18 higher.)	-	102 (4 Randomised Controlled Trials [RCTs])	⊕◯◯◯ Very low[b,c,e,f]	There is no significant difference in favour of the use of *tDCS for gait* speed endurance in post-stroke patients.
Cadence evaluated with: *Gait analysis*	-	SMD is **0.67 SD higher.** (0.6 lower than 1.93 higher.)	-	40 (2 Randomised Controlled Trials [RCTs])	⊕◯◯◯ Very low[b,c,e]	There is no significant difference in favour of the use of *tDCS for gait* speed cadence in post-stroke patients.

The risk in the intervention group (and its 95% confidence interval) is based on the risk assumed in the comparison group and the **relative effect** of the intervention (and its 95% confidence interval).

CI: Confidence Interval ; **SMD:** Standardised Mean Difference

GRADE Working Group Degrees of Evidence
High certainty: We are very confident that the true effect is close to the effect estimate.
Moderate certainty: We have moderate confidence in the effect estimate: the true effect is likely to be close to the effect estimate, but there is a possibility that it could be substantially different.
Low certainty: We have limited confidence in the effect estimate: the actual effect may be substantially different from the effect estimate.
Very low certainty: We have very low confidence in the effect estimate: The actual effect is likely to be substantially different from the effect estimate.

Explanations

a. No studies have performed concealed allocation.
b. Low sample size (<400)
c. The CI exceeds the threshold between recommending and not recommending treatment.
d. 2 of the 4 included studies did not perform concealed allocation.
e. 1 study did not perform concealed allocation.
f. CIs do not overlap. High heterogeneity.

Summary of results: Dong et al. [21]

tDCS combined with physiotherapy compared to placebo to improve gait in post-stroke patients

Patient or population: improving gait in post-stroke patients
Configuration:
Intervention: *tDCS* combined with physiotherapy
Comparison: placebo

Outcomes	Anticipated absolute effects* (95% CI)		Relative effect (95% CI)	Number of participants (studies)	Certainty of evidence (GRADE)	Comments
	**Placebo risk**	**Risk with *tDCS* combined with physiotherapy**				
Walking speed was evaluated with: *10MWT*	The average walking speed was **-0.93**	MD **0.93 lower** (2.68 lower to 0.82 higher.)	-	79 (4 Randomised Controlled Trials (RCTs))	⊕◯◯◯ Very low[a,b,c]	There is no significant difference in favour of the use of *tDCS for gait* speed improvement.
Functional mobility is assessed with: *TUG*	The average functional mobility was **-0.92**	MD **2.18 lower** (4.51 lower than 0.15 higher.)	-	130 (5 Randomised Controlled Trials [RCTs])	⊕◯◯◯ Very low[b,c,d]	No significant differences are evident in favour of the use of *tDCS for* the improvement of functional gait mobility.
Functional mobility is assessed with: *FAC*	The average functional mobility was **0.7**	MD **0.34 higher.** (0.14 lower than 0.82 higher).	-	122 (5 Randomised Controlled Trials [RCTs])	⊕◯◯◯ Very low[b,c,e,f]	No significant differences are evident in favour of the use of *tDCS for* the improvement of functional gait mobility.
Motor function is assessed with: *FMA-LE*	The mean motor function was **1.85**	MD **0.43 lower** (1.7 lower to 0.84 higher.)	-	115 (4 Randomised Controlled Trials [RCTs])	⊕◯◯◯ Very low[b,c,g]	No significant differences were found in favour of the use of *tDCS for* the improvement of lower limb motor function.
Gait resistance was tested with: *6MWT*	The average march endurance was **27.65**	MD **2.55 lower** (18.34 lower to 13.23 higher.)	-	101 (4 Randomised Controlled Trials [RCTs])	⊕⊕◯◯ Low[b,c,h]	No significant differences are evident in favour of the use of *tDCS for* the improvement of gait endurance.

The risk in the intervention group (and its 95% confidence interval) is based on the risk assumed in the comparison group and the **relative effect** of the intervention (and its 95% confidence interval).

CI: Confidence Interval; **MD:** Mean Difference

Summary of results: Dong et al. [21]

tDCS combined with physiotherapy compared to placebo to improve gait in post-stroke patients

Patient or population: improving gait in post-stroke patients
Configuration:
Intervention: *tDCS* combined with physiotherapy
Comparison: placebo

Outcomes	**Anticipated absolute effects*** (95% CI)		Relative effect (95% CI)	Number of participants (studies)	Certainty of evidence (GRADE)	Comments
	**Placebo risk**	**Risk with *tDCS* combined with physiotherapy**				

GRADE Working Group Degrees of Evidence
High certainty: We are very confident that the true effect is close to the effect estimate.
Moderate certainty: We have moderate confidence in the effect estimate: the true effect is likely to be close to the effect estimate, but there is a possibility that it could be substantially different.
Low certainty: We have limited confidence in the effect estimate: the actual effect may be substantially different from the effect estimate.
Very low certainty: We have very low confidence in the effect estimate: The actual effect is likely to be substantially different from the effect estimate.

Explanations

a. 3 of the included studies have a moderate risk of bias in randomisation and concealed allocation.
b. Low sample size (<400)
c. The CI exceeds the threshold between recommending and not recommending treatment.
d. 4 of the included studies were at moderate risk of bias in randomisation and concealed allocation.
e. 4 of the included studies were at high and moderate risk of bias in randomisation and concealed allocation.
f. Low overlap in CI. High heterogeneity.
g. 2 of the included studies are at moderate risk of allocation bias and concealed allocation.
h. 1 study had a high risk of bias in concealed allocation, but this was not significant enough to downgrade the result by one level.

Summary of results: Navarro-López et al. [15]

tDCS combined with physiotherapy compared to a placebo for improving gait in post-stroke patients

Patient or population: improving gait in post-stroke patients
Configuration:
Intervention: *tDCS* combined with physiotherapy
Comparison: placebo

Outcomes	**Anticipated absolute effects*** (95% CI)		Relative effect (95% CI)	Number of participants (studies)	Certainty of evidence (GRADE)	Comments
	**Placebo risk**	**Risk with *tDCS* combined with physiotherapy**				
Gait speed was evaluated with: *10MWT* and quantitative systems.	Two studies showed improvements in both groups, but no significant improvements were found between groups.			53 (3 Randomised Controlled Trials [RCTs])	⨁⨁◯◯ Low[a,b]	Evidence suggests that *tDCS*, combined with physiotherapy, does not increase walking speed.
Functional mobility is assessed with: *FAC*	2 studies evaluated FAC data. None found significant differences between groups.			35 (2 Randomised Controlled Trials [RCTs])	⨁⨁◯◯ Low[b,c]	Evidence suggests that *tDCS*, combined with physiotherapy, does not increase functional mobility.
Functional mobility is assessed with: *TUG*	3 studies found improvements in both groups at the end of treatment. Only 1 found significant differences between groups (p=0.018).			44 (3 Randomised Controlled Trials [RCTs])	⨁⨁◯◯ Low[b,c]	Evidence suggests that *tDCS*,, combined with physiotherapy, does not increase functional mobility.
Functional mobility is assessed with: *RMI*	Only 1 study evaluated data for this outcome. It showed no significant differences between groups.			11 (1 RCT (randomised controlled experiment))	⨁⨁◯◯ Low[a,b]	Evidence suggests that *tDCS*,, combined with physiotherapy, does not increase functional mobility.
Motor Functionality is assessed with: *FMA-LE*	1 study evaluated the data for this outcome. This study found significant differences from the control group (P=0.023).			24 (1 RCT (randomised controlled experiment))	⨁⨁◯◯ Low[a,b]	Evidence suggests that *tDCS* combined with physiotherapy results in a slight increase in functional mobility.
Functional mobility is assessed with: *Tinetti*	1 study showed significant differences between the two groups. Only 1 study found significant differences between groups (p=0.049) at 4 weeks of treatment.			45 (2 Randomised Controlled Trials [RCTs])	⨁⨁◯◯ Low[b,d]	Evidence suggests that *tDCS*, combined with physiotherapy, does not increase functional mobility.
Gait resistance was tested with: *6MWT*	1 study showed improvements in both groups. 2 studies found significant differences between groups (p=0.038).			89 (3 Randomised Controlled Trials (RCTs))	⨁⨁◯◯ Low[b,e]	Evidence suggests that *tDCS* combined with physiotherapy results in a slight increase in gait endurance.

Summary of results: Navarro-López et al. [15]

tDCS combined with physiotherapy compared to a placebo for improving gait in post-stroke patients

Patient or population: improving gait in post-stroke patients
Configuration:
Intervention: *tDCS* combined with physiotherapy
Comparison: placebo

Outcomes	**Anticipated absolute effects*** (95% CI)		Relative effect (95% CI)	Number of participants (studies)	Certainty of evidence (GRADE)	Comments
	**Placebo risk**	**Risk with *tDCS* combined with physiotherapy**				

The risk in the intervention group (and its 95% confidence interval) is based on the risk assumed in the comparison group and the **relative effect** of the intervention (and its 95% confidence interval).

CI: Confidence Interval

GRADE Working Group Degrees of Evidence
High certainty: We are very confident that the true effect is close to the effect estimate.
Moderate certainty: We have moderate confidence in the effect estimate: the true effect is likely to be close to the effect estimate, but there is a possibility that it could be substantially different.
Low certainty: We have limited confidence in the effect estimate: the actual effect may be substantially different from the effect estimate.
Very low certainty: We have very low confidence in the effect estimate: The actual effect is likely to be substantially different from the effect estimate.

Explanations

a. This study was at high and moderate risk of bias in the blinding of participants, therapists, and assessors.
b. Low sample size (<400).
c. Included studies were at high and moderate risk of bias in randomisation, concealed allocation and blinding of participants, therapists and assessors.
d. One study had a moderate risk of bias.
e. 2 studies were at moderate risk of bias.

Summary of results: Veldema et al. [22]

tDCS applying anode on the ipsilesional hemisphere compared to placebo to improve gait in post-stroke patients

Patient or population: improving gait in post-stroke patients
Configuration:
Intervention: *tDCS* applying anode on ipsilesional hemisphere
Comparison: placebo

Outcomes	**Anticipated absolute effects*** (95% CI)		Relative effect (95% CI)	Number of participants (studies)	Certainty of evidence (GRADE)	Comments
	**Placebo risk**	**Risk with *tDCS* applying anode on ipsilesional hemisphere**				
Functionality of lower limbs is assessed with: Balance, gait and motor function		The effect size is **0.34 higher.** (0.38 lower than 1.08 higher).	-	195 (7 Randomised Controlled Trials [RCTs])	⨁◯◯◯ Very low[a,b,c]	*tDCS* applying anode over the ipsilesional hemisphere may result in little to no difference in lower limb functionality.

The risk in the intervention group (and its 95% confidence interval) is based on the risk assumed in the comparison group and the **relative effect** of the intervention (and its 95% confidence interval).

CI: Confidence Interval

GRADE Working Group Degrees of Evidence
High certainty: We are very confident that the true effect is close to the effect estimate.
Moderate certainty: We have moderate confidence in the effect estimate: the true effect is likely to be close to the effect estimate, but there is a possibility that it could be substantially different.
Low certainty: We have limited confidence in the effect estimate: the actual effect may be substantially different from the effect estimate.
Very low certainty: We have very low confidence in the effect estimate: The actual effect is likely to be substantially different from the effect estimate.

Explanations

a. The included studies are at risk of blinding bias.
b. The CI exceeds the threshold between recommending treatment and not recommending treatment.
c. Low sample size (<400)

Summary of results: Veldema et al. [22]

tDCS or *tACS* bilaterally compared to placebo for improving gait in post-stroke patients

Patient or population: improving gait in post-stroke patients
Configuration:
Intervention: *tDCS* or *tACS* bilaterally
Comparison: placebo

Outcomes	**Anticipated absolute effects*** (95% CI)		Relative effect (95% CI)	Number of participants (studies)	Certainty of evidence (GRADE)	Comments
	**Placebo risk**	**Risk with *tDCS* or *tACS* bilaterally**				
Functionality of lower limbs is assessed with: Balance, gait and motor function		The effect size is **1.15 higher.** (0.27 higher. to 2.04 higher.)	-	135 (5 Randomised Controlled Trials [RCTs])	⊕◯◯◯ Very low[a,b,c]	*tDCS* bilaterally could result in a large increase in lower limb functionality.

The risk in the intervention group (and its 95% confidence interval) is based on the risk assumed in the comparison group and the **relative effect** of the intervention (and its 95% confidence interval).

CI: Confidence Interval

GRADE Working Group Degrees of Evidence
High certainty: We are very confident that the true effect is close to the effect estimate.
Moderate certainty: We have moderate confidence in the effect estimate: the true effect is likely to be close to the effect estimate, but there is a possibility that it could be substantially different.
Low certainty: We have limited confidence in the effect estimate: the actual effect may be substantially different from the effect estimate.
Very low certainty: We have very low confidence in the effect estimate: The actual effect is likely to be substantially different from the effect estimate.

Explanations

a. The included studies are at risk of blinding bias.
b. Heterogeneity of 80%.
c. Low sample size (<400)

Summary of results: Veldema et al. [22]

tDCS in the contralesional hemisphere using the anode compared to placebo to improve gait in post-stroke patients

Patient or population: improving gait in post-stroke patients
Configuration:
Intervention: *tDCS* in the contralesional hemisphere, applying the anode
Comparison: placebo

Outcomes	**Anticipated absolute effects*** (95% CI)		Relative effect (95% CI)	Number of participants (studies)	Certainty of evidence (GRADE)	Comments
	**Placebo risk**	**Risk with *tDCS* in the contralesional hemisphere with anode application**				
Lower limb functionality is assessed with: Balance, gait and motor function		The effect size is **0.06 higher.** (0.65 lower to 0.78 higher).	-	15 (1 RCT (Randomised Controlled Trial))	⊕◯◯◯ Very low[a,b,c]	The evidence is very unclear on the effect of *tDCS* on the contralesional hemisphere applying the anode to lower limb functionality.

The risk in the intervention group (and its 95% confidence interval) is based on the risk assumed in the comparison group and the **relative effect** of the intervention (and its 95% confidence interval).

CI: Confidence Interval

GRADE Working Group Degrees of Evidence
High certainty: We are very confident that the true effect is close to the effect estimate.
Moderate certainty: We have moderate confidence in the effect estimate: the true effect is likely to be close to the effect estimate, but there is a possibility that it could be substantially different.
Low certainty: We have limited confidence in the effect estimate: the actual effect may be substantially different from the effect estimate.
Very low certainty: We have very low confidence in the effect estimate: The actual effect is likely to be substantially different from the effect estimate.

Explanations

a. The included study is at risk of blinding bias.
b. Low sample size (<400)
c. The CI exceeds the threshold between recommending treatment and not recommending treatment.

Summary of results: Veldema et al. [22]

tDCS in the contralesional hemisphere with cathode application compared to placebo to improve gait in post-stroke patients

Patient or population: improving gait in post-stroke patients
Configuration:
Intervention: *tDCS* on the contralesional hemisphere**,** applying the cathode
Comparison: placebo

Outcomes	Anticipated absolute effects* (95% CI)		Relative effect (95% CI)	Number of participants (studies)	Certainty of evidence (GRADE)	Comments
	**Placebo risk**	**Risk with *tDCS in the* contralesional hemisphere with cathode application**				
Functionality of lower limbs is assessed with: Balance, gait and motor function		The effect size is**3.11 higher.** (1.97 higher. to 4.24 higher.)	-	60 (1 RCT (Randomised Controlled Trial))	⊕⊕◯◯ Low[a,b]	*tDCS* in the contralesional hemisphere applying the cathode could result in a large increase in lower limb functionality.

The risk in the intervention group (and its 95% confidence interval) is based on the risk assumed in the comparison group and the **relative effect** of the intervention (and its 95% confidence interval).

CI: Confidence Interval

GRADE Working Group Degrees of Evidence
High certainty: We are very confident that the true effect is close to the effect estimate.
Moderate certainty: We have moderate confidence in the effect estimate: the true effect is likely to be close to the effect estimate, but there is a possibility that it could be substantially different.
Low certainty: We have limited confidence in the effect estimate: the actual effect may be substantially different from the effect estimate.
Very low certainty: We have very low confidence in the effect estimate: The actual effect is likely to be substantially different from the effect estimate.

Explanations

a. The included study is at risk of blinding bias.
b. Low sample size (<400)

Summary of results: Veldema et al. [22]

tDCS combining anode in SM1 and cathode in cerebellum compared to placebo to improve gait in post-stroke patients

Patient or population: improving gait in post-stroke patients
Configuration:
Intervention: *tDCS* combining **the** anode in SM1 and **the** cathode in **the** cerebellum
Comparison: placebo

Outcomes	**Anticipated absolute effects*** (95% CI)		Relative effect (95% CI)	Number of participants (studies)	Certainty of evidence (GRADE)	Comments
	**Placebo risk**	**Risk with *tDCS* combining anode in SM1 and cathode in cerebellum**				
Lower limb functionality is assessed with: Balance, gait, and motor function		The effect size is **0.03 lower** (0.66 lower to 0.67 higher.)	-	30 (1 RCT (Randomised Controlled Trial))	⊕◯◯◯ Very low[a,b,c]	The evidence is very unclear on the effect of *tDCS* combining the anode in SM1 and the cathode in the cerebellum on lower limb functionality.

The risk in the intervention group (and its 95% confidence interval) is based on the risk assumed in the comparison group and the **relative effect** of the intervention (and its 95% confidence interval).

CI: Confidence Interval

GRADE Working Group Degrees of Evidence
High certainty: We are very confident that the true effect is close to the effect estimate.
Moderate certainty: We have moderate confidence in the effect estimate: the true effect is likely to be close to the effect estimate, but there is a possibility that it could be substantially different.
Low certainty: We have limited confidence in the effect estimate: the actual effect may be substantially different from the effect estimate.
Very low certainty: We have very low confidence in the effect estimate: The actual effect is likely to be substantially different from the effect estimate.

Explanations

a. The included study has a risk of bias in blinding.
b. Low sample size (<400)
c. The CI exceeds the threshold between recommending treatment and not recommending treatment.

Summary of results: Veldema et al. [22]

tDCS combining the anode in SM1 and the cathode in the cerebellum compared to placebo to improve gait in post-stroke patients

Patient or population: improving gait in post-stroke patients
Configuration:
Intervention: *tDCS* combining **the** anode in SM1 and **the** cathode in **the** cerebellum
Comparison: placebo

Outcomes	**Anticipated absolute effects**[*] (95% CI)		Relative effect (95% CI)	Number of participants (studies)	Certainty of evidence (GRADE)	Comments
	**Placebo risk**	**Risk with *tDCS* combining anode in SM1 and cathode in cerebellum**				
Lower limb functionality is assessed with: Balance, gait and motor function		The effect size is **0.03 lower** (0.66 lower to 0.67 higher.)	-	30 (1 RCT (Randomised Controlled Trial))	⊕◯◯◯ Very low[a,b,c]	The evidence is very unclear on the effect of *tDCS* combining the anode in SM1 and the cathode in the cerebellum on lower limb function.

The risk in the intervention group (and its 95% confidence interval) is based on the risk assumed in the comparison group and the **relative effect** of the intervention (and its 95% confidence interval).

CI: Confidence Interval

GRADE Working Group Degrees of Evidence
High certainty: We are very confident that the true effect is close to the effect estimate.
Moderate certainty: We have moderate confidence in the effect estimate: the true effect is likely to be close to the effect estimate, but there is a possibility that it could be substantially different.
Low certainty: We have limited confidence in the effect estimate: the actual effect may be substantially different from the effect estimate.
Very low certainty: We have very low confidence in the effect estimate: The actual effect is likely to be substantially different from the effect estimate.

Explanations

a. The included study is at risk of blinding bias.
b. Low sample size (<400)
c. The CI exceeds the threshold between recommending treatment and not recommending treatment.

Summary of results: Corominas-Teruel et al. [23]

tDCS compared to placebo for improving gait in post-stroke patients

Patient or population: improving gait in post-stroke patients
Configuration:
Intervention: *tDCS*
Comparison: placebo

Outcomes	**Anticipated absolute effects*** (95% CI)		Relative effect (95% CI)	Number of participants (studies)	Certainty of evidence (GRADE)	Comments
	**Placebo risk**	**Risk with *tDCS***				
Walking speed	Results are shown as effect size (cohen's d): 3 studies showed no effect (< 0.20). 5 studies had small effect sizes of 0.2, 0.23, 0.39, 0.4 and 0.42. Finally, one study showed a large effect size of 1.33.			684 (14 Randomised Controlled Trials [RCTs])	⊕◯◯◯ Very low[a,b,c,d]	Evidence suggests that *tDCS* combined with physiotherapy does not increase walking speed.
Functional mobility is assessed with*: TUG*	Results are shown as effect size (cohen's d): 3 studies showed no effect (<0.20). 3 studies showed a small effect of 0.4 and 0.5.			237 (8 Randomised Controlled Trials [RCTs])	⊕◯◯◯ Very low[a,b,c,d]	Evidence suggests that *tDCS* combined with physiotherapy, does not increase functional mobility.
Functional mobility is assessed with: *Tinetti*	Results are shown as effect size (cohen's d): 1 study reported a moderate effect size of 0.7, while 2 studies showed large effect sizes of 0.94 and 1.21.			150 (3 Randomised Controlled Trials [RCTs])	⊕◯◯◯ Very low[a,b,c,d]	Evidence suggests that *tDCS* combined with physiotherapy, does not increase functional mobility.
Walking resistance	Results are shown as effect size (cohen's d): We found that 3 studies had a small effect of 0.3, 0.28, and 0.28. 1 study had a moderate effect of 0.6 and 1 study had a large effect of 1.1. while 5 studies had insufficient data to calculate the effect size.			572 (10 Randomised Controlled Trials [RCTs])	⊕◯◯◯ Very low[a,b,c,d]	Evidence suggests that *tDCS* combined with physiotherapy does not increase gait endurance.

The risk in the intervention group (and its 95% confidence interval) is based on the risk assumed in the comparison group and the **relative effect** of the intervention (and its 95% confidence interval).

CI: Confidence Interval

GRADE Working Group Degrees of Evidence
High certainty: We are very confident that the true effect is close to the effect estimate.
Moderate certainty: We have moderate confidence in the effect estimate: the true effect is likely to be close to the effect estimate, but there is a possibility that it could be substantially different.
Low certainty: We have limited confidence in the effect estimate: the actual effect may be substantially different from the effect estimate.
Very low certainty: We have very low confidence in the effect estimate: The actual effect is likely to be substantially different from the effect estimate.

Explanations

a. The included studies are at risk of blinding bias.
b. Narrative explanations are considered inaccurate.
c. Low sample size (<400)
d. The direction of effect varies across studies.

Summary of results: Bressi et al. [24]

tDCS in combination with a gait-assisted robot compared to a placebo for improving gait in post-stroke patients

Patient or population: improving gait in post-stroke patients
Configuration:
Intervention: *tDCS* in combination with a gait-assisted walking robot
Comparison: placebo

Outcomes	**Anticipated absolute effects*** (95% CI)		Relative effect (95% CI)	Number of participants (studies)	Certainty of evidence (GRADE)	Comments
	**Placebo risk**	**Risk with *tDCS* in combination with a gait-assisted robot**				
Walking speed was evaluated with: *10MWT*	1 study showed no significant difference, although it did favour the treatment group (p=0.19). 2 studies also found no difference in favour of the use of *tDCS*.			48 (3 Randomised Controlled Trials [RCTs])	⊕◯◯◯ Very low[a,b,c]	Evidence suggests that *tDCS*, combined with physiotherapy does not increase walking speed.
Functional mobility is assessed with: *TUG*	1 study showed significant differences between groups (p=0.066).			8 (1 RCT (Randomised Controlled Trial))	⊕◯◯◯ Very low[a,b,c]	Evidence suggests that *tDCS*, combined with physiotherapy, does not increase functional mobility.
Functional mobility is assessed with: *RMI*	1 study found no significant improvement.			20 (1 RCT (Randomised Controlled Trial))	⊕⊕◯◯ Low[b,c]	Evidence suggests that *tDCS*, combined with physiotherapy, does not increase functional mobility.
Functional mobility is assessed with: *FAC*	1 study showed significant differences between groups (p=0.026). Another study found no significant improvement between groups. 1 study found significant differences in the group receiving anode stimulation during follow-up (p=0.024).			48 (3 Randomised Controlled Trials (RCTs))	⊕◯◯◯ Very low[a,b,c]	Evidence suggests that *tDCS*, combined with physiotherapy, does not increase functional mobility.
Gait resistance was tested with: *6MWT*	1 study found no significant improvement.			20 (1 RCT (Randomised Controlled Trial))	⊕⊕◯◯ Low[b,c]	Evidence suggests that *tDCS* ,combined with physiotherapy, does not increase gait endurance.
Motor function is assessed with: *FMA-LE*	1 study found no significant improvement.			20 (1 RCT (Randomised Controlled Trial))	⊕⊕◯◯ Low[b,c]	Evidence suggests that *tDCS* ,combined with physiotherapy, does not increase motor function.

Summary of results: Bressi et al. [24]

tDCS in combination with a gait-assisted robot compared to a placebo for improving gait in post-stroke patients

Patient or population: improving gait in post-stroke patients
Configuration:
Intervention: *tDCS* in combination with a gait-assisted walking robot
Comparison: placebo

Outcomes	Anticipated absolute effects* (95% CI)		Relative effect (95% CI)	Number of participants (studies)	Certainty of evidence (GRADE)	Comments
	**Placebo risk**	**Risk with *tDCS* in combination with a gait-assisted robot**				
Muscle strength is assessed with: *MRC*	1 study found no significant improvement.			20 (1 RCT (Randomised Controlled Trial))	⊕⊕◯◯ Low[b,c]	Evidence suggests that *tDCS* combined with physiotherapy does not increase muscle strength.

The risk in the intervention group (and its 95% confidence interval) is based on the risk assumed in the comparison group and the **relative effect** of the intervention (and its 95% confidence interval).

CI: Confidence Interval

GRADE Working Group Degrees of Evidence
High certainty: We are very confident that the true effect is close to the effect estimate.
Moderate certainty: We have moderate confidence in the effect estimate: the true effect is likely to be close to the effect estimate, but there is a possibility that it could be substantially different.
Low certainty: We have limited confidence in the effect estimate: the actual effect may be substantially different from the effect estimate.
Very low certainty: We have very low confidence in the effect estimate: The actual effect is likely to be substantially different from the effect estimate.

Explanations

a. Risk of bias in the randomisation process in a study
b. Low sample size (<400)
c. Narrative explanations are considered inaccurate.
